# Neofunctionalization of a duplicate hatching enzyme gene during the evolution of teleost fishes

**DOI:** 10.1186/s12862-014-0221-0

**Published:** 2014-10-19

**Authors:** Kaori Sano, Mari Kawaguchi, Satoshi Watanabe, Shigeki Yasumasu

**Affiliations:** 1 Department of Chemistry, Faculty of Science, Josai University, 1-1 Keyakidai, Sakado, Saitama 350-0295 Japan; 2 Department of Materials and Life Sciences, Faculty of Science and Technology, Sophia University, 7-1 Kioi-cho, Chiyoda-ku, Tokyo, 102-8554 Japan; 3 National Research Institute of Aquaculture, Fisheries Research Agency, 422-1 Nakatsuhamaura, Minami-ise, Mie 516-0193 Japan

**Keywords:** Neofunctionalization, Hatching enzyme, Evolution of protein function, Egg envelope digestion

## Abstract

**Background:**

Duplication and subsequent neofunctionalization of the teleostean hatching enzyme gene occurred in the common ancestor of Euteleostei and Otocephala, producing two genes belonging to different phylogenetic clades (clade I and II). In euteleosts, the clade I enzyme inherited the activity of the ancestral enzyme of swelling the egg envelope by cleavage of the N-terminal region of egg envelope proteins. The clade II enzyme gained two specific cleavage sites, N-ZPd and mid-ZPd but lost the ancestral activity. Thus, euteleostean clade II enzymes assumed a new function; solubilization of the egg envelope by the cooperative action with clade I enzyme. However, in Otocephala, the clade II gene was lost during evolution. Consequently, in a late group of Otocephala, only the clade I enzyme is present to swell the egg envelope. We evaluated the egg envelope digestion properties of clade I and II enzymes in Gonorynchiformes, an early diverging group of Otocephala, using milkfish, and compared their digestion with those of other fishes. Finally, we propose a hypothesis of the neofunctionalization process.

**Results:**

The milkfish clade II enzyme cleaved N-ZPd but not mid-ZPd, and did not cause solubilization of the egg envelope. We conclude that neofunctionalization is incomplete in the otocephalan clade II enzymes. Comparison of clade I and clade II enzyme characteristics implies that the specificity of the clade II enzymes gradually changed during evolution after the duplication event, and that a change in substrate was required for the addition of the mid-ZPd site and loss of activity at the N-terminal region.

**Conclusions:**

We infer the process of neofunctionalization of the clade II enzyme after duplication of the gene. The ancestral clade II gene gained N-ZPd cleavage activity in the common ancestral lineage of the Euteleostei and Otocephala. Subsequently, acquisition of cleavage activity at the mid-ZPd site and loss of cleavage activity in the N-terminal region occurred during the evolution of Euteleostei, but not of Otocephala. The clade II enzyme provides an example of the development of a neofunctional gene for which the substrate, the egg envelope protein, has adapted to a gradual change in the specificity of the corresponding enzyme.

## Background

Gene duplication is one of the driving forces of evolution. In the most cases, one of the duplicated genes is subsequently lost, but in some cases, both genes are retained and diverged, leading to shared original function (subfunctionalization). Alternatively, one of the genes acquires a new function (neofunctionalization) [1,2]. To understand this process, there is a need to study the evolution of the protein and its function. However, progress in this field has been slow because of the complexity of protein structures and their interactions. In this study, we propose a model for understanding neofunctionalization of duplicated genes using hatching enzymes and egg envelopes.

**Figure 1:**
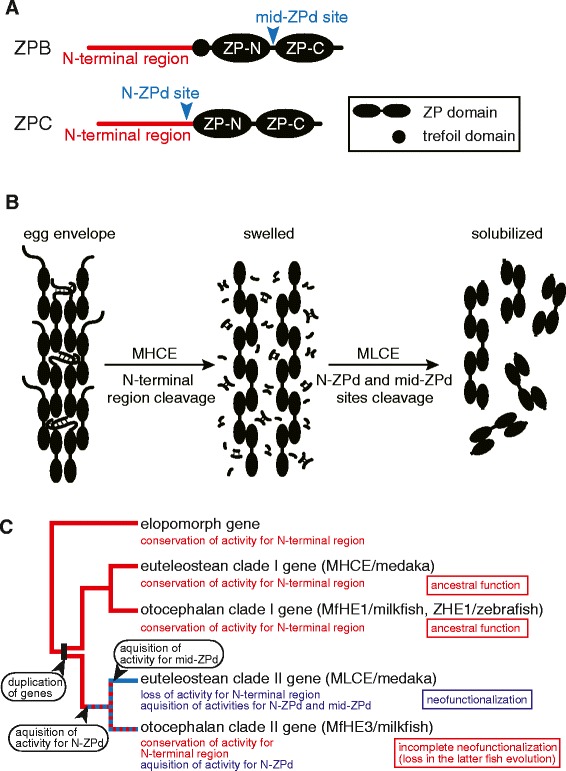
**The egg envelope digestion manner of medaka hatching enzymes and the process of neofunctionalization of teleostean hatching enzyme genes.** **(A)** A schematic diagram of ZP protein structure and names of cleavage sites. **(B)** Egg envelope digestion mechanism of medaka hatching enzyme, MHCE and MLCE. **(C)** The process of neofunctionalization of teleostean hatching enzyme genes inferred by the present study.The names of enzymes analyzed in the present study are in parenthesis.

Teleostean egg envelopes, the substrate for hatching enzymes, are constructed of two types of glycoproteins, the ZP proteins ZPB and ZPC related to mammalian zona pellucida subunits ZP1 and ZP3, respectively. ZP proteins commonly possess an N-terminal region and Zona Pellucida (ZP) domain, which consists of two folds (ZP-N and ZP-C) connected by a linker sequence [3]. In addition, ZPB proteins have a trefoil domain between the N-terminal region and the ZP domain (Figure 1A). The macromolecular architecture of the vertebrate egg envelope develops as follows (Figure 1A). The ZP domains are polymerized to each other to form long filaments by inter-molecular non-covalent interactions between the subdomains [4-6]. The N-terminal regions loop out from the filaments [3], and tie or bunch the filamentous structures [7]. In fish, the ε-(γ-glutamyl) lysine isopeptide cross-links (Glu-Lys cross-links) are formed between the filamentous structures at fertilization. This makes the inter-filament interactions tight, and turns the egg envelope into a hard and tough structure. At the time of hatching, the fertilized egg envelopes are digested by a hatching enzyme that is secreted by the embryo.

Hatching enzyme, which belongs to the astacin metalloproteinase family [8,9], is found in fish, amphibians, birds, reptiles, and mammals [7,10-13]. In teleosts, we have cloned 67 hatching enzyme genes from 27 species, and elucidated their evolutionary pathway [14,15]. The majority of fishes have several hatching enzyme genes in their genomes, suggesting that there were several duplication events of hatching enzyme genes during evolution of the fish lineage [16].

Teleostean fishes are classified into three subdivisions, Elopomorpha, Otocephala, and Euteleostei [17]. Phylogenetic analysis of hatching enzyme genes suggests the elopomorph genes form a monophyletic clade. Seven hatching enzyme cDNAs with 85–95% amino acid identity were cloned from the Japanese eel *Anguilla japonica* (Elopomorpha) [18]. However, these isozymes were functionally the same in terms of their role in egg envelope digestion [19]. Gene diversification has occurred in the common ancestor of Otocephala and Euteleostei, such that members of these subdivisions possess two types of genes, belonging to two clades, clade I and clade II. Moreover, during the evolution of Otocephala, the clade II gene of Otophysi has been lost [15].

Analysis of the egg envelope digestion mechanism by hatching enzymes has been studied in a number of fish species. The primitive mechanism of teleostean hatching was predicted using Japanese eel. A single type of hatching enzyme digests the N-terminal region of ZPB and ZPC into short peptide fragments. Its cleavage leads to cancelation of the inter-molecular Glu-Lys cross-links of ZP proteins and renders the egg envelope swollen and softened [19].

In contrast, medaka *Oryzias latipes* (Euteleostei) hatch by the cooperative action of two hatching enzymes, clade I and II enzymes (Figure 1B). The clade I enzyme named MHCE (medaka high choriolytic enzyme) cleaves the N-terminal regions of ZPB, where many of the Glu-Lys cross-links are located [20], into short peptide fragments, thereby swelling the egg envelope in a manner similar to the Japanese eel single enzyme [7]. The clade II enzyme named MLCE (medaka low choriolytic enzyme) cleaves two specific sites on the swollen egg envelope, termed the mid-ZPd and N-ZPd sites. The mid-ZPd site is located at the intervening sequence between two subdomains, ZP-N and ZP-C, in the ZP domain of ZPB [3,7,21,22], whereas the N-ZPd site is located at the N-terminus of the ZP domain of ZPC (Figure 1A). The cleavage of the two sites causes disruption of ZP domain filamentous structures, thereby solubilizing the swollen egg envelope. The position of respective cleavage sites of the clade I and clade II enzymes on ZP proteins is highly conserved in euteleostean fishes [22].

Based on the egg envelope digestion manner, it is thought that the ancestral function of the fish hatching enzyme was to induce swelling of the egg envelope by cleaving the N-terminal region and breaking the intermolecular Glu-Lys cross-links. The Japanese eel hatching enzyme gene and the clade I genes of Euteleostei have inherited this ancestral function. In contrast, euteleostean clade II genes are thought to have developed a new function, the solubilization of the swollen egg envelope by cleavage at two specific sites. As described above, the clade II gene was lost during evolution of the otocephalan lineage. Zebrafish *Danio rerio* belong to the Otophysi, a late group of Otocephala, and possess only the clade I enzyme (ZHE1), the activity of which is analogous to that of Japanese eel hatching enzyme and MHCE [23]. From an evolutionary perspective, the egg envelope digestion mechanism of otophysans appears to have reverted to the ancestral form.

We evaluated the mechanisms of egg envelope digestion in Gonorynchiformes, an early diverging group of Otocephala, using milkfish *Chanos chanos*. This species possesses both clade I and II genes. We compared their digestion properties with those of other fishes and inferred the neofunctionalization process of the clade II hatching enzyme as follows. After diversification of the gene, the ancestral clade II gene has gained N-ZPd cleavage activity in the lineage of common ancestors of Euteleostei and Otocephala. Subsequently, acquisition of cleavage activity at the mid-ZPd site and loss of cleavage activity in the N-terminal region has occurred during the evolution of Euteleostei, but not Otocephala. Therefore, the addition of the two cleavage sites is presumed to be the result of independent evolutionary events (Figure 1C). Finally, by comparing their egg envelope cleavage specificities, we propose a hypothesis to explain why the clade II gene was lost in the otocephalan lineage and how the euteleostean clade II gene acquired a new function.

## Results and discussion

### Characterization of recombinant hatching enzymes

**Figure 2:**
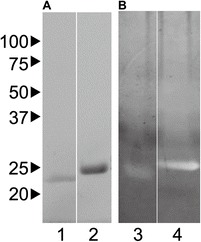
**Generation of recombinant milkfish hatching enzymes. (A)** SDS-PAGE patterns of recombinant milkfish hatching enzymes, rMfHE1 (lane 1) and rMfHE3 (lane 2), stained with Coomassie Brilliant Blue. **(B)** Casein zymography patterns of rMfHE1 (lane 3) and rMfHE3 (lane 4). The numbers on the left represent the size of the molecular markers.

Two clade I enzymes (MfHE1 and MfHE2) and a clade II enzyme (MfHE3) have been cloned from milkfish [15]. The active recombinant enzymes were successfully refolded from the inclusion bodies, although the refolding efficiency of recombinant MfHE2 (rMfHE2) was much lower than that of rMfHE1 and rMfHE3. SDS-PAGE of the rMfHE1 and rMfHE3 yielded bands at 23 and 24.5 kDa, respectively, which is consistent with values calculated from mature enzyme sequences of cDNAs and His-tag (24076.69 for rMfHE1 and 24129.73 for rMfHE3, Figure 2A). The results of zymography revealed that the mobility of bands having caseinolytic activity corresponded to that of the CBB stained bands (Figure 2B). The specific caseinolytic activities of the nickel column-purified rMfHE1 and rMfHE3 were 26.8 and 14.2 Δ_280_ · min^−1^ · mg protein^−1^, respectively. These values are similar to those of other fish hatching enzymes (30.7 Δ_280_ · min^−1^ · mg protein^−1^ for ZHE1, 27.3 Δ_280_ · min^−1^ · mg protein^−1^ for MHCE, and 18.4 Δ_280_ · min^−1^ · mg protein^−1^ for MLCE) [19]. Thus, we concluded that recombinant enzymes were of sufficient quality to use in the egg envelope digestion experiments.

### Structural changes in the egg envelope induced by MfHE1 and MfHE3

**Figure 3:**
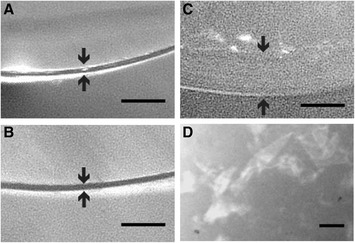
**Morphological changes in the egg envelope caused by hatching enzymes.** Isolated egg envelopes **(A)** were digested by rMfHE1 **(B)** and rMfHE3 **(C)**. The egg envelope digested by rMfHE3 was crushed with forceps and the broken pieces were observed **(D)**. Scale bars: 50 μm.

First, we observed the morphological changes in the milkfish fertilized egg envelope digested by recombinant hatching enzymes (rMfHE1 and rMfHE3) using an optical microscope. The MfHE1-treated envelope was softened and became slightly thicker than the intact envelope (Figure 3A, B). In contrast, treatment with MfHE3 caused dramatic swelling in the egg envelope (Figure 3C), and became quite fragile and easily broken into gelatinous pieces by crushing with forceps (Figure 3D). The morphology of the egg envelopes treated by a mixture of MfHE1 and MfHE3 was similar to that of the MfHE3-treated envelope (data not shown), suggesting the two enzymes do not have a synergistic effect.

### Determination of the MfHE1 and MfHE3 cleavage sites in unfertilized egg envelopes

The egg envelope becomes hardened after fertilization by formation of ε-(γ-glutamyl) lysine isopeptide cross-links [24,25]. Because these cross-links make it difficult to clearly determine the cleavage sites of the egg envelope, unfertilized egg envelopes were used as substrates. The cleavage sites of egg envelope proteins by rMfHEs were determined by sequencing the N-terminal of the digests and matching the sequences with those of milkfish ZP proteins deduced from cDNAs cloned previously (MfZPB, AB759543; MfZPCa, AB759544; MfZPCb, AB759545). Because MfZPCa and MfZPCb share similar amino acid sequences, only the MfZPCa sequence is shown.

**Figure 4:**
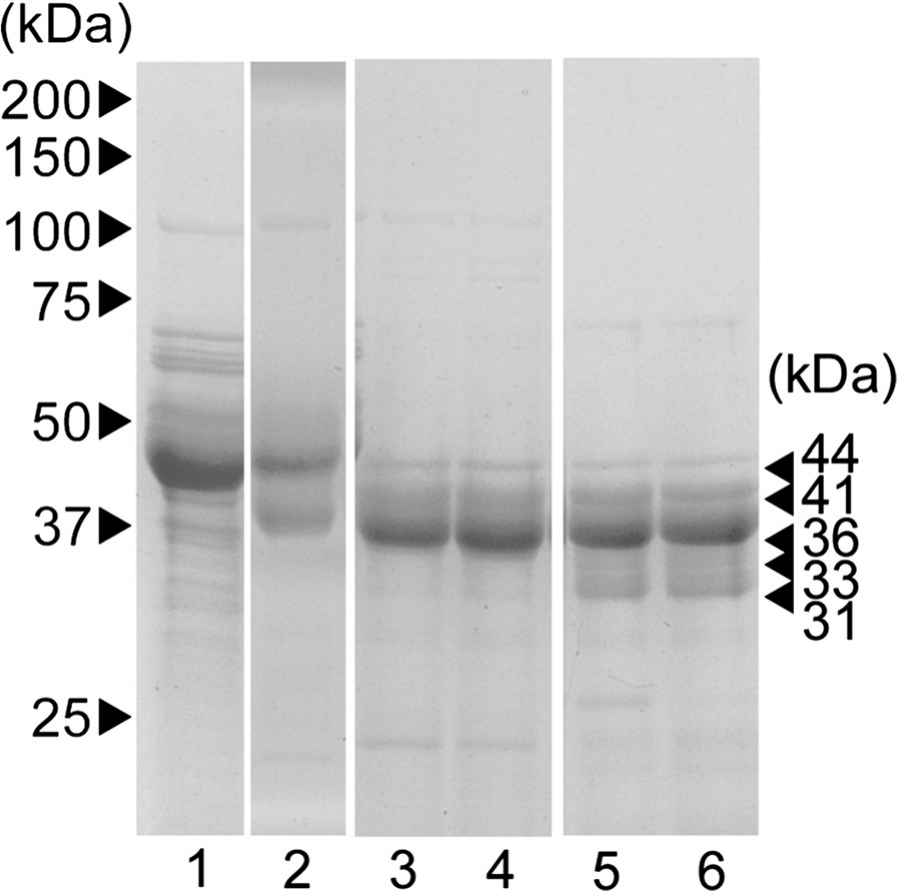
**SDS-PAGE of unfertilized egg envelopes and their digests following treatment with MfHEs.** Egg envelopes isolated from unfertilized eggs of the milkfish (lane 1) were digested by rMfHE1 for 15, 30 or 60 min (lane 2–4), or rMfHE3 for 30 or 60 min (lane 5, 6). The numbers on the right represent the sizes of the major bands. The numbers on the left represent the sizes of the molecular markers.

**Figure 5:**
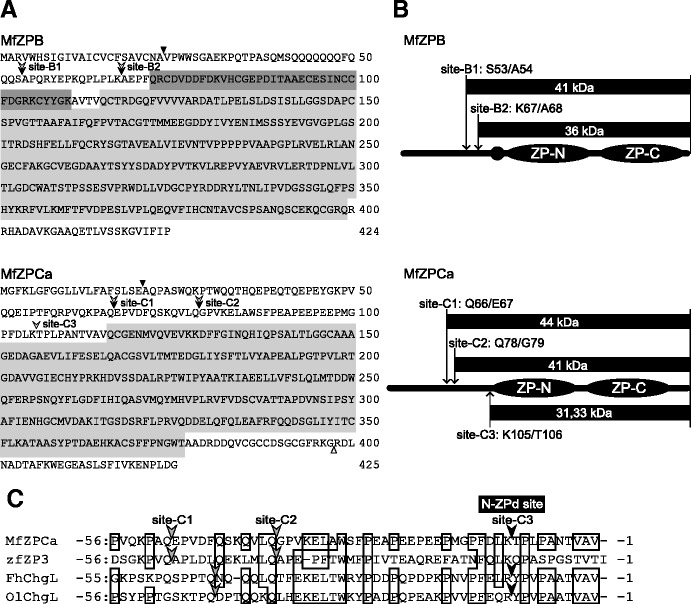
**The cleavage sites of MfHE1 and MfHE3, and schematic diagram of predicted products. (A)** amino acid sequences predicted from cDNA of milkfish ZPB and ZPCa, and the cleavage sites of MfHEs. The black and grey arrowheads indicate the cleavage sites of MfHE1 and MfHE3, respectively. The ZP domains and trefoil domain are shown in light and dark gray boxes, respectively. The black triangles are putative cleavage sites of the signal peptidase. The white triangle represents C-terminal processing sites. **(B)** Schematic diagrams of MfZPB and MfZPCa, and the cleavage sites of MfHE1 and MfHE3. The predicted molecular sizes of the products produced by MfHE1 and MfHE3 are shown as black boxes. ZP-N and ZP-C indicate the two subdomains in the ZP domain. The small circle in MfZPB indicates the trefoil domain. **(C)** Alignment of the amino acid sequences of ZPC around the N-ZPd site of milkfish (MfZPCa), zebrafish (zfZP3), killifish (FhChgL), and medaka (OlChgL). Identical residues are boxed and dashes represent gaps. The gray arrowheads indicate the cleavage sites for each fish hatching enzyme in this region. The black arrow heads indicate the N-ZPd sites specifically cleaved by clade II enzymes, MfHE3, FLCE, and MLCE. The amino acid sequence is numbered from the N-terminus of the ZP domain.

SDS-PAGE of the unfertilized egg envelope yielded a major 44 kDa band and several minor bands (Figure 4 lane 1). SDS-PAGE of the MfHE1 digest after a 15 min incubation yielded two major bands at 44 and 41 kDa (Figure 4, lane 2). The sequencing of the 44-kDa band revealed that it was a mixture of two peptides. The major sequence, VPWWSGA, corresponded to the N-terminal of undigested MfZPB (Figure 5A). Another minor sequence, EPVDF, was found from E_67_ in MfZPCa, and the cleavage sites were Q_66_/E_67_ (site-C1, Figure 5A). The sequence of the 41-kDa product was also a mixture of two sequences, APQRYE and GPVKELA. The former sequence was found in MfZPB from A_54_, and the cleavage site was S_53_/A_54_ (site-B1, Figure 5A). The latter sequence was found in MfZPCa from G_79_, and the cleavage sites were Q_78_/G_79_ (site-C2, Figure 5A). After incubation for 30 min, the 44-kDa band weakened, and a 36-kDa band developed (Figure 4, lane 3). The N-terminal sequences obtained from the 41-kDa band were the same as after the 15 min incubation. The N-terminal sequence of the 36-kDa band corresponded to the sequence of MfZPB from A_69_ and the cleavage site was K_68_/A_69_ (site-B2, Figure 5A). A longer incubation period (60 min) did not alter the SDS pattern and the cleavage sites are the same as those of 30 min-incubation (Figure 4, lane 4), suggesting that the digests obtained from the 30 min incubation were the final products and there was no further cleavage by MfHE1.

In summary, two MfHE1 cleavage sites in MfZPB (site-B1 and site-B2) and two sites in MfZPCa (site-C1 and site-C2) were located in the N-terminal region of MfZPB and MfZPCa (Figure 5A, B). The cleavage sites of MfHE2 and MfHE1 were identical (data not shown), suggesting that these two clade I enzymes act on the egg envelope in the same manner.

The SDS-PAGE patterns of the MfHE3 digests were similar, but not identical to those of MfHE1. SDS-PAGE of MfHE3 digests yielded four bands at 41, 36, 33, and 31 kDa following a 30 min incubation (Figure 4, lane 5). The cleavage sites determined from the first two products were identical to those for MfHE1, i.e., 41 kDa for site-C2 and site-B1, and 36 kDa for site-B2 (Figure 5A). Therefore, sites-B1, −B2, and -C2 were in common between MfHE1 and MfHE3. Conversely, latter two bands (33 and 31 kDa) represented MfHE3 specific cleavage sites. Sequencing of these bands yielded the same sequence (TPLPAN) found in MfZPCa from T_106_, and the cleavage sites (site-C3) were at K_105_/T_106_ (Figure 5A). The difference in molecular mass between the two digests is thought to be caused by differences in their sugar moieties. Interestingly, the position of site-C3 corresponds to the N-ZPd site in euteleosts, medaka, and killifish *Fundulus heteroclitus* (Figure 5C). Thus, MfHE3 cleaved three sites located in the N-terminal region in common with MfHE1, and one unique site, site-C3, named N-ZPd, in MfZPCa (Figure 5A, B). There was no difference between the SDS-PAGE patterns from the 60 (Figure 4, lane 6) and 30 min incubations, suggesting that the digests obtained following a 30 min incubation represented the final digestion products of MfHE3.

### Determination of the MfHE1 and MfHE3 cleavage sites in fertilized egg envelopes

**Figure 6:**
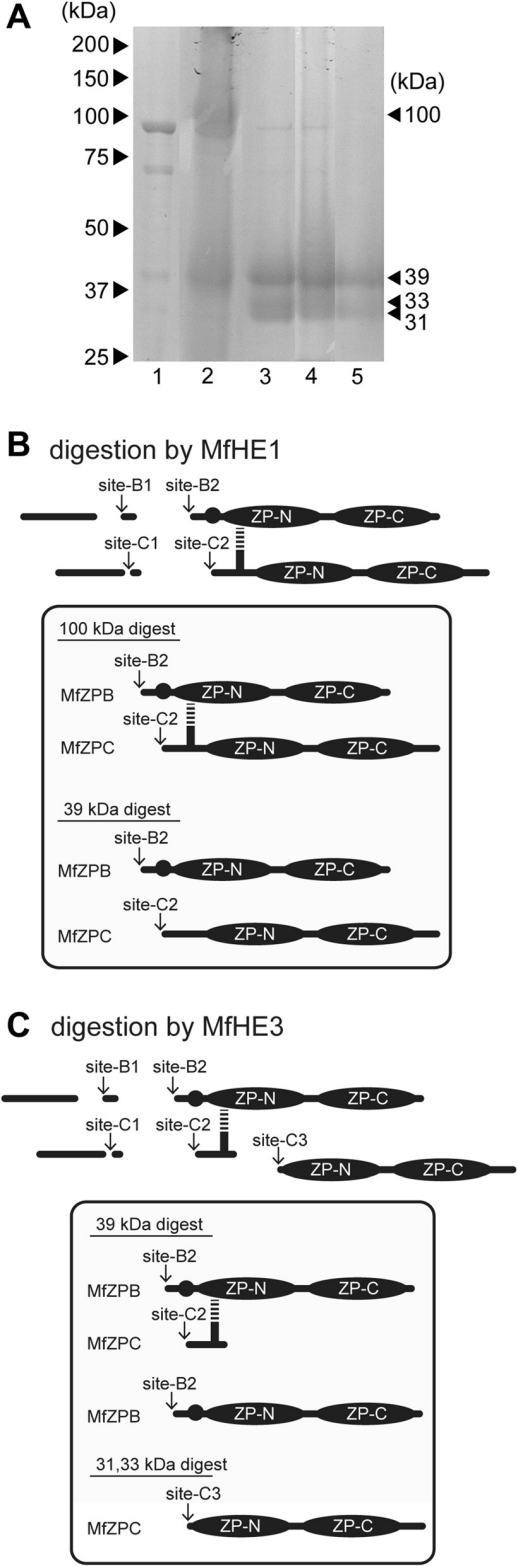
**Digestion of fertilized egg envelopes by MfHEs. (A)** SDS-PAGE patterns of fertilized egg envelope digests. Fertilized egg envelopes are extracted by SDS as a control (lane 1), and digested by rMfHE1 (lane 2), rMfHE3 (lane 3), or a mixture of rMfHE1 and rMfHE3 (lane 4). Egg envelopes after hatching are extracted by SDS (lane 5). The numbers on the right represent the sizes of the major band of digests. The numbers on the left represent the sizes of the molecular markers. **(B, C)** Schematic diagrams of the predicted digestion mechanism of fertilized egg envelopes by MfHE1 and MfHE3. The arrows indicate the position of the cleavage sites of MfHE1 and MfHE3, respectively. Broken line represent the predicted position of intermolecular ε-(γ-glutamyl) lysine cross-links between MfZPB and MfZPCa.

The fertilized egg envelope provides a substrate for hatching enzymes at the time of hatching. The fertilized egg envelope was digested by rMfHE1 and rMfHE3 to determine the location of the cleavage sites. The fertilized eggs were not soluble in SDS (Figure 6A, lane 1), but became soluble following digestion by MfHE1 or MfHE3. SDS-PAGE of the MfHE1 digests yielded a broad band at 39 kDa and a band at ~100 kDa (Figure 6A, lane 2). The two cleavage sites determined from the N-terminal amino acid sequences of the 39-kDa band were site-B2 and site-C1. The molecular weights from the cleavage sites to the C-terminus of ZP proteins estimated from the respective cDNAs were 36,281.33 and 36,347.47, respectively (Figure 5A). Our results suggest that the 39-kDa band was a mixture of two peptides spanning the region from site-B2 to the end of the ZP domain of MfZPB (B2-MfZPB) and from site-C1 to the C-terminal processing site of MfZPCa (C1-MfZPCa) (Figure 6B). The cleavage sites determined from the 100 kDa band included sites-B2, -C1, and -C2 (Figures 5A and 6B). Two of the three cleavage sites obtained from the 100 kDa band were identical to those of the 39 kDa band. Thus, we concluded that the 100 kDa band was a dimer or trimer of the digests of the 39 kDa band (Figure 6B). Together, our observations suggest that the 100 kDa band is a complex formed by B2-MfZPB, C1-MfZPCa, and C2-MfZPCa, and presumably linked by ε-(γ-glutamyl) lysine bonds.

SDS-PAGE of fertilized egg envelopes digested by MfHE3 yielded three bands at 39, 33, and 31 kDa (Figure 6A, lane 3). The cleavage sites determined from the 39 kDa band included sites-B2, -C1, and -C2, which are identical to those of the 100 kDa band from MfHE1 (Figure 5A). The sequences determined for the 33 and 31 kDa bands were identical, and the cleavage site was located at site-C3 (also known as the N-ZPd site) which corresponds to the unique site for MfHE3 determined by the digestion of unfertilized egg envelopes (Figure 5A, B, Figure 6C). The 100 kDa band observed in the MfHE1 digest was not observed in the MfHE3 digest. Four of the five cleavage sites of MfHE3 were shared with MfHE1. Therefore, cleavage of the N-ZPd site (site-C3) by MfHE3 resulted in further digestion of the 100 kDa band into a 39 and 31/33 kDa band, leading to the removal of the cross-link between the 39 kDa peptides (Figure 6C). It is reasonable to conclude that one of the two amino acid residues responsible for ε-(γ-glutamyl) lysine cross-link formation was present in the region between site-C2 and site-C3 in MfZPCa. Indeed, two lysine residues that are candidates for the acceptor site of the cross-links are present in this region (Figure 5A, C). Consequently, cleavage of the N-ZPd site by MfHE3 cleaved a small section of the peptides, including the cross-links (Figure 6C). As a result, the tight bindings between the ZP proteins are eliminated.

The same SDS-PAGE pattern was obtained from the mixture of MfHE1 and MfHE3, and from the egg envelopes after natural hatching (Figure 6A, lane 4 and 5). Their cleavage sites were consistent with those obtained from the MfHE3 digests. Our results are also consistent with morphological observations. MfHE1 slightly swelled and softened the egg envelope by cleaving the N-terminal region into fine fragments, while MfHE3 caused markedly swelling of the egg envelope by cleaving both the N-terminal region and the N-ZPd site leading to complete elimination of the cross-links (Figure 3). In the following section, we compare the egg envelope digestion process and the substrate specificity of milkfish hatching enzymes with those of other fish hatching enzymes to trace the neofunctionarization process. The characters of fish hatching enzymes discussed in the following sections are listed in Table 1.

**Table 1:** **The list of hatching enzymes used in the present study**

**Fish**	**Classification**	**Enzyme**	**Clade**	**Cleavage sites**	**Reference**
Milkfish	Gonorynchiformes, Otocephala	MfHE1	I	N-terminal region	Present study
		MfHE3	II	N-terminal region and N-ZPd	Present study
Medaka	Beloniformes, Euteleostei	MHCE	I	N-terminal region	[7,37]
		MLCE	II	N-ZPd and mid-ZPd	[7,38]
Zebrafish	Cypriniformes, Otocephala	ZHE1	I	N-terminal region	[23]

### Why was the otocephalan clade II gene lost during evolution to Otophysi?

**Figure 7:**
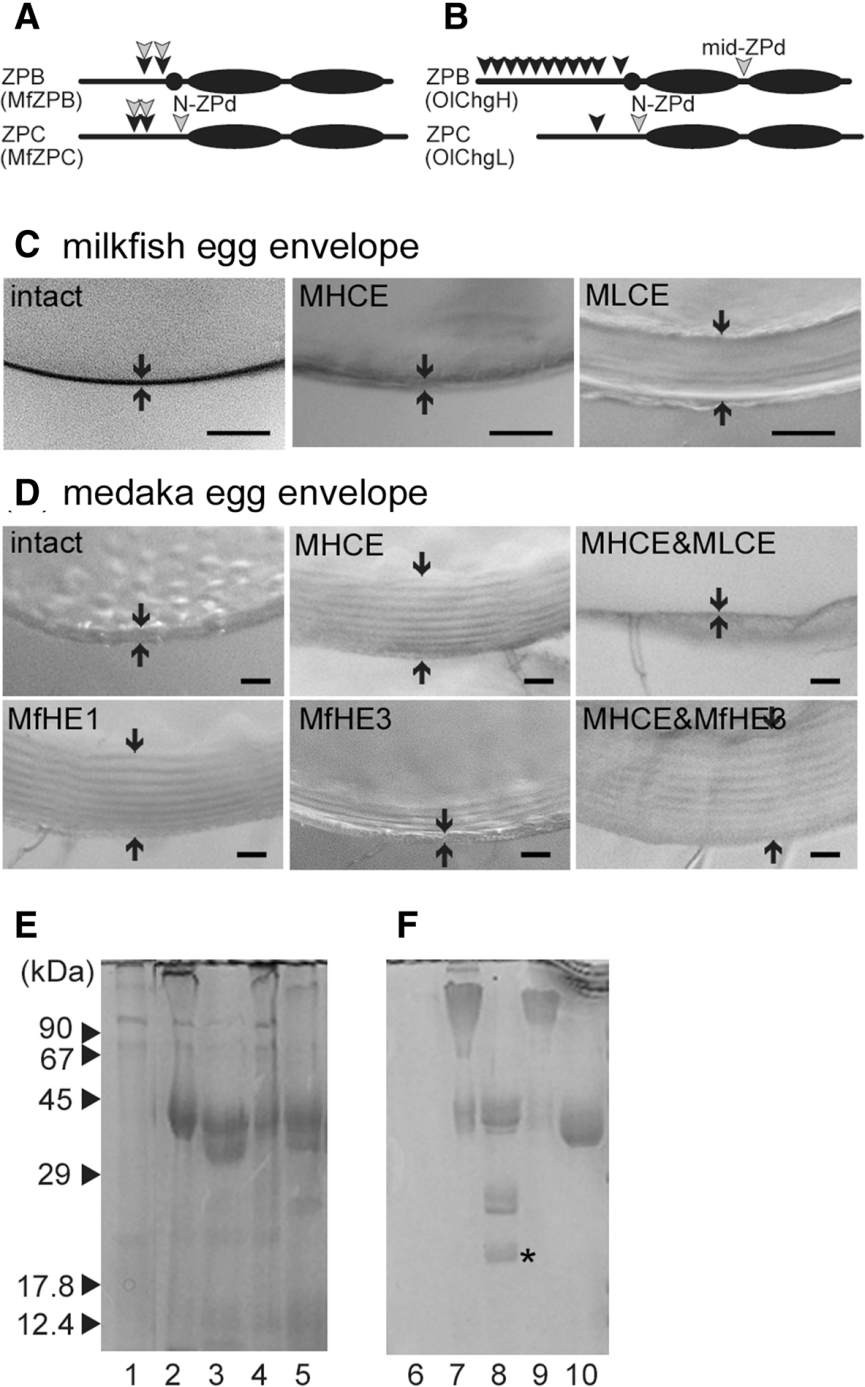
**Cross-species digestion experiment exchanging hatching enzyme-egg envelope combinations between milkfish and medaka.** Schematic diagrams of the cleavage sites of milkfish **(A)** and medaka hatching enzymes **(B)** on the egg envelope proteins, ZPB and ZPC. Black and gray arrowheads indicate cleavage sites of clade I and clade II enzyme, respectively. **(C)** The structural changes in the milkfish egg envelope caused by medaka hatching enzymes. The intact egg envelope, the MHCE-treated egg envelope, and the MLCE-treated egg envelope are shown from left to right. **(D)** The structural changes in the medaka egg envelope caused by medaka and milkfish hatching enzymes. The intact egg envelope, MHCE-treated egg envelope, MLCE-treated MHCE-swollen egg envelope are shown in the upper photos. The MfHE1-treated egg envelope, MfHE3-treated egg envelope, and MfHE3-treated MHCE-swollen egg envelope are shown in the lower photos. **(E)** SDS-PAGE patterns of the digests of milkfish egg envelope. Undigested fertilized egg envelope (lane 1), egg envelope digested by MHCE (lane 2), by MLCE (lane 3), by MfHE1 (lane 4), and by MfHE3 (lane 5). **(F)** SDS-PAGE of the digests of medaka egg envelope. Undigested fertilized egg envelope (lane 6), egg envelope digested by MHCE (lane 7) and MfHE1 (lane 9), MHCE-swollen egg envelope digested by MLCE (lane 8) and by MfHE3 (lane 10). The asterisk indicates the 15 and 16 kDa products produced by cleavage of the mid-ZPd site. Numbers on the left represent the sizes of molecular markers.

We compared the egg envelope digestion manner between milkfish and medaka hatching enzymes (Figure 7A, B). Both clade I enzymes are thought to have conserved the ancestral function of cleaving the N-terminal region, resulting in swelling of the egg envelope. However, the clade II enzymes differ between the two groups. The otocephalan clade II enzyme does not exhibit cleavage activity at the mid-ZPd site, where a euteleostean clade II enzyme specifically cleaves. Furthermore, it does cleave the N-terminal region. Thus, the process of neofunctionalization does not appear to be complete for the otocephalan clade II gene, MfHE3. However, the otocephalan clade II enzyme rendered the egg envelope more swollen and softened than the clade I enzymes because it had cleavage activity at the N-ZPd site (Figure 7A). Thus, the otocephalan clade II enzyme is more effective than the clade I enzyme, and was therefore conserved in the early diverging group of Otocephala, Clupeiformes and Gonorynchiformes. However, the possession of two enzymes for egg envelope digestion would be dispensable for hatching, so otophysans were able to persist despite the loss of one of the enzymes. Embryos are able to rupture the softened egg envelope by moving, and thereby hatch. The egg envelope digestion of fish in the more recently diverged groups (Otophysi) have reverted to the ancestral manner, namely the swelling of the egg envelope by a single enzyme, cf. zebrafish hatching enzyme, ZHE1 [23].

Another factor to consider is the evolution of egg envelope genes. We focused our evaluation on the tissue of synthesis of egg envelope proteins, ZP proteins. The teleostean ZP proteins were originally synthesized by oocytes in the ovary. The zp genes expressed in both the liver and the ovary of the common ancestor of Otocephala and Euteleostei were produced by gene duplication [26,27]. A number of euteleosts synthesize the major components of the egg envelope in the liver, whereas the otocephalan liver expressing genes have disappeared in the evolutionary lineage to Ostariophysi, which synthesize ZP proteins in the ovary. Therefore, the evolution of the egg envelope digestion system, from a single enzyme to a two-enzymes system, has nearly overlapped with a change in the expression sites of zp genes. The liver is the largest organ in the body, and has the capacity to synthesize a large amount of protein, including yolk proteins (vitellogenins) in fish and frogs [28,29]. In fact, when the thicknesses of the egg envelopes were compared, the egg envelopes of euteleosts tended to be thicker than those of otophysans [26,30]. It is conceivable that hatching from a thicker envelope requires a more efficient digestion system involving two enzymes. Conversely, swelling and softening of the egg envelope by a single enzyme may be sufficient for hatching of otocephalan embryos because of the thin egg envelope. It is possible that the duplication of the zp genes followed by a switch of the expression site is closely related to the evolutional event of loss of the clade II gene in otocephalan lineage.

In the following section, we develop a hypothesis to explain the conditions under which a gene acquires a new function by comparing MLCE and MfHE3, representatives of the euteleostean and otocephalan clade II enzymes, respectively.

### Exchange of hatching enzyme-egg envelope combination

#### Conservation of activity of the clade I enzyme

To infer the mechanism by which egg envelope digestion by hatching enzymes was conserved during evolution, the egg envelope-enzyme combinations were altered between milkfish and medaka. First, we focused on the clade I enzymes, MfHE1 and MHCE. MHCE slightly swelled and softened milkfish egg envelope, as did MfHE1 (Figures 7C and 3B). The SDS-PAGE patterns of the digests were similar (Figure 7E lane 2, 4), and N-terminal sequences of the digests from MHCE were the same as those from MfHE1. Similarly, digestion of medaka egg envelope by MfHE1 resulted in morphological changes that were similar to those caused by MHCE (Figure 7D). Additionally, the N-terminal sequences of the digests corresponded to the sequences obtained from MHCE digests (Figure 7F lane 7, 9). Therefore, the xenogeneic combination of MHCE and MfHE1 cleaved the same sites as the allogeneic combination, suggesting conservation of substrate specificity in clade I enzymes during evolution.

#### Acquisition of a new function by the clade II enzyme

The pattern of digestion by the xenogeneic combination of clade II enzymes, MfHE3 and MLCE, differed from that of the allogeneic combination. MLCE did not digest the intact medaka egg envelope to any significant extent, but did dissolve the swollen egg envelope following digestion by MHCE (Figure 7D). The dissolved egg envelope yielded a 37 kDa band caused by cleavage of the N-ZPd site in ZPC and two low molecular bands at 17 and 16 kDa produced by cleavage mid-ZPd in ZPB (Figure 7B, F lane 8) [7]. Interestingly, MLCE caused significant swelling of the milkfish egg envelope, as did MfHE3 (Figures 3C and 7C). Sequence analysis of the digests revealed that MLCE cleavage sites in the milkfish egg envelope were the same as those of MfHE3. These observations suggest that MLCE cleaves the N-terminal region and the N-ZPd site but not the mid-ZPd site of the milkfish egg envelope.

The single action of MfHE3 had little effect on the intact medaka egg envelope. However, swelling was observed in the inner surface of the egg envelope (Figure 7D). These observations suggest that MfHE3 is inefficient at digesting the N-terminal region. When MfHE3 acts on the MHCE-swollen egg envelope, the egg envelope undergoes further swelling (Figure 7D). This structural change was similar to that caused by MfHE3 in the milkfish egg envelope (Figure 3C). Cleavage of the N-ZPd site was confirmed by sequencing of the broad 36 kDa band of MHCE-MfHE3 digests (Figure 7F lane 10). The low molecular digests produced by the cleavage of the mid-ZPd site were not observed in the MHCE-MfHE3 digest, suggesting that MfHE3 did not have the activity to cleave the mid-ZPd site in the medaka egg envelope.

### Inferring changes in the substrate specificity of clade I and II enzymes during evolution

#### The cleavage activity of N-terminal region; ancestral function

We further investigated the efficiency of cleavage at sites in the N-terminal regions using synthetic peptide substrates that were designed based on the cleavage sites of two euteleosts, medaka [7], and killifish [22], and two otocephalans, zebrafish [23], and milkfish (present study, site-C1 and -C2) (Table 2). In addition to milkfish and medaka hatching enzymes, we used zebrafish hatching enzyme, ZHE1 [23], as an additional representative of the otocephalan clade I enzyme.

**Table 2:** **Specific activity of enzymes examined with peptides designed from N-terminal regions**

	**Sequence of substrate**	**Species**	**Specific activities (μmol · mg enzyme** ^**−1**^ **· 30 min** ^**−1**^ **)**				
			MfHE1	MHCE	ZHE1	MfHE3	MLCE
1	NPQVPQ↓YPSKPQ	Medaka	1.55	9.76	1.01	0.02	0.05
2	NPSYPQ↓NPSYPQ	Medaka	4.87	16.1	2.11	0.002	0.08
3	PPSKPQ↓YPNPQT	Killifish	ND	0.66	ND	0.018	ND
4	QPQTPS↓YPQQPQ	Killifish	1.05	2.46	0.42	0.039	0.57
5	PLPVR↓VEEVV	Zebrafish	ND	0.02	0.44	ND	ND
6	KLMLQ↓APEPF	Zebraish	1.87	3.36	1.85	1.28	2.33
7	TVQQS↓DYLIK	Zebrafish	2.93	2.74	3.06	1.19	1.00
8	FQQQS↓APQRY	Milkfish	8.07	1.36	2.68	0.28	3.52
9	PLPLK↓AEPFQ	Milkfish	4.27	2.30	6.38	0.79	ND
10	KQVLQ↓GPVKE	Milkfish	0.57	ND	0.20	0.03	ND

**Figure 8:**
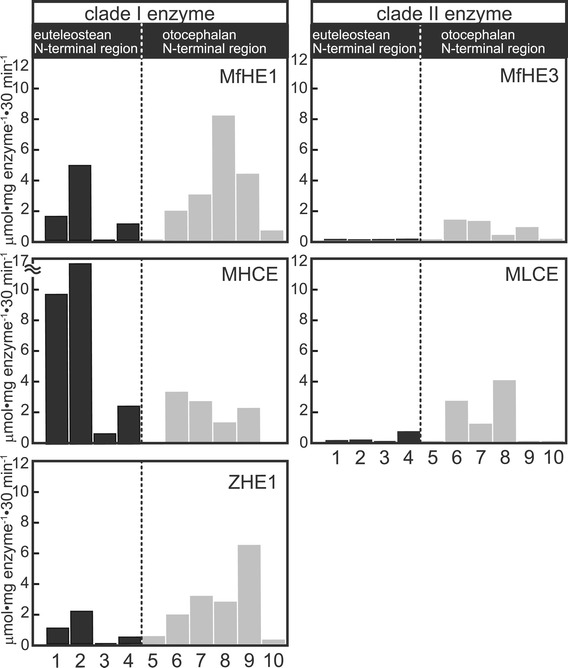
**The specific activities of clade I and clade II enzymes toward synthetic peptide substrates designed from the cleavage sites at the N-terminal region of ZP proteins of Euteleostei and Otocephala.** Black bars represent Euteleoste (1,2: medaka, 3,4: killifish), and gray bars represent Otocephala (5, 6, 7: zebrafish, 8, 9, 10: milkfish). The specific activities of three clade I enzymes (MfHE1, MHCE and ZHE1) and two clade II enzymes (MfHE3 and MLCE) were examined using 10 peptides, whose sequences were shown in Table 2.

Arrows indicate cleavage sites. The numbers on the left correspond to the substrate numbers in Figure 8.

When the specific activities toward the substrates of otocephalan N-terminal region were compared, all of the enzymes tended to cleave their peptides with sufficient specific activity, although MLCE only partially cleaved the peptides (three of the six, Figure 8). However, the specificity toward peptides designed from the euteleostean N-terminal region differed between clade I and II enzymes. The clade I enzymes MfHE1, MHCE, and ZHE1 cleaved most of the substrates designed from euteleostean N-terminal region, whereas the clade II enzymes MfHE3 and MLCE were inefficient at cleaving these peptides (Figure 8). Thus, the substrate specificity was similar within the clade I and within II enzyme groups, suggesting that the substrate specificities of these two clades have been conserved during evolution. The difference between clade I and II enzymes in terms of their specificity for the euteleostean N-terminal regions, was consistent with the results in the cross-species digestion experiments in which MfHE3 and MLCE caused swelling of the intact milkfish egg envelope, but not the intact medaka egg envelope.

The ZPB N-terminal regions in many euteleosts, but not otocepharans, contain a characteristic three amino acid repeat motif, known as the Pro-Gln-X repeat [26]. This implies that the N-terminal region sequence of the ZP protein has changed significantly along the evolutionary pathway to Euteleostei. Given that both euteleostean and otocephalan clade I enzymes cleave at the Glu-X bonds in the repeat sequence, we hypothesize that the euteleostean N-terminal region has changed within the range of cleavage specificity of clade I enzyme to maintain the ancestral function, swelling egg envelope. However, this change has led to loss of cleavage activity by clade II enzymes. Our results suggest that a change in the substrate sequences in evolutionary pathway to the Euteleostei would be important for the loss of ancestral function of the clade II gene.

#### Two cleavage sites, N-ZPd and mid-ZPd, acquire a new function

***The cleavage activity of N-ZPd sites***

**Figure 9:**
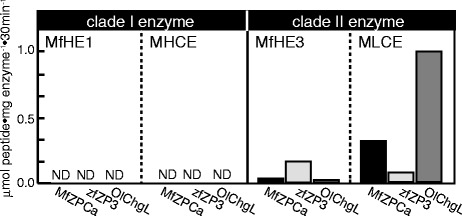
**The specific activities of clade I and clade II enzymes of milkfish (MfHE1 and MfHE3) and medaka (MHCE and MLCE) toward synthetic peptide substrates.** The peptides are designed from the N-ZPd site of ZPC of milkfish (MfZPC), zebrafish (zfZP3), and medaka (OlChgL). The sequences are shown in Table 3.

Both Euteleostei and Otocephala shared a specific cleavage site (N-ZPd) for the clade II enzyme in their respective ZP proteins. We compared the cleavage activities toward the N-ZPd site using peptide substrates designed from milkfish and medaka, and the corresponding position in zebrafish (Table 3). The clade II enzymes MfHE3 and MLCE cleaved not only their own N-ZPd site but also the peptides designed from other fish N-ZPd sites (Figure 9). Conversely, clade I enzymes were inefficient at cleaving the N-ZPd sites. These results were consistent with observations in the egg envelope digestion experiment.

**Table 3:** **Specific activity of enzymes examined with peptides designed from N-ZPd sites**

	**Sequence**	**Specific activities (μmol · mg enzyme** ^**−1**^ **· 30 min** ^**−1**^ **)**			
		MfHE1	MHCE	MfHE3	MLCE
MfZPCa	PFDLK↓TPLPA	ND	ND	0.03	0.31
zfZP3	NFQLK↓QPASP	ND	ND	0.16	0.07
OlChgL	VPFEQR↓YPVPA	ND	ND	0.02	1.00

Arrows indicate cleavage sites.

***The cleavage activity of mid-ZPd sites***

**Figure 10:**
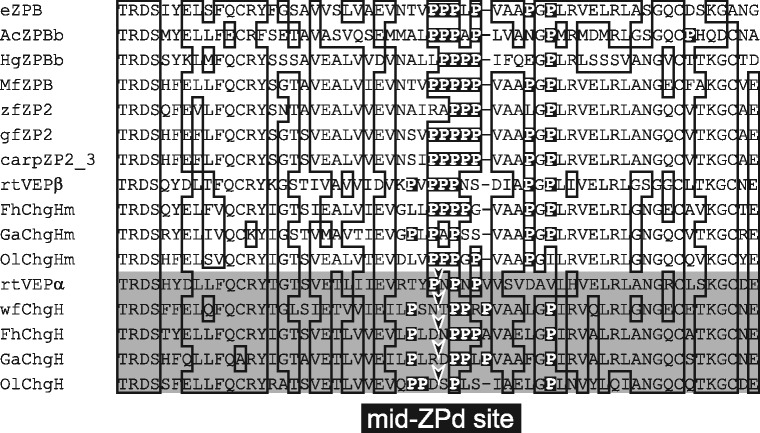
**Alignment of amino acid sequences of ZPB around the mid-ZPd sites.** The sequences of euteleostean ZPB are highlighted by the gray box. Arrowheads indicate the mid-ZPd cleavage sites. The proline residues are shown by white letters in a black box. The name of the ZPB genes and their source are as follows. eZPB, Japanese eel; AcZPBb, Japanese anchovy *Engraulis japonicus*; HgZPBb, Pacific herring *Clupea pallasii*; MfZPB, milkfish; zfZP2, zebrafish; gfZP2, goldfish *Carassius auratus auratus*; carpZP2_3, carp *Cyprinus carpio*; rtVEPα and rtVEPβ, rainbow trout *Oncorhynchus mykiss*; wfChgH, winter flounder *Pseudopleuronectes americanus*; FhChgH and FhChgHm, killifish; GaChgH and GaChgHm, three-spined stickleback *Gasterosteus aculeatus*, and OlChgH and OlChgHm, medaka.

The cross-species egg envelope digestion experiments revealed that cleavage of the N-ZPd site by MfHE3 did not solubilize the medaka egg envelope (Figure 7D, F). Therefore, acquisition of the mid-ZPd site during evolution was likely critical for the establishment of the euteleostean two-enzymes system. We compared the amino acid sequences around the mid-ZPd site among the ZPBs of various fish species (Figure 10). Euteleostean egg envelopes are constructed by two ZPBs corresponding to the precursor proteins, choriogenin H (ChgH) and choriogenin Hm (ChgHm) [31]. The euteleostean clade II enzymes cleave the mid-ZPd site of ChgH, but not ChgHm [22]. The alignment of amino acid sequences, including the mid-ZPd site, is shown in Figure 10. In elopomorph and otocephalan sequences, the proline cluster (located between −1 to +4 of the cleavage site) was well conserved. Additionally, the sequences of ChgHm of Euteleostei contained a cluster of three prolines spanning the sites corresponding to the mid-ZPd (located between −1 to +2 of the cleavage site). Conversely, the euteleostean ChgH did not have a proline cluster spanning the cleavage site, and the proline residues at the +1 and −1 sites were frequently substituted by other amino acid residues (Figure 10). There are no prior reports regarding proteases that cleave between Pro-Pro bonds (MEROPS, the peptidase database http://merops.sanger.ac.uk/). These findings suggest that the addition of the cleavage site in the mid-ZPd is accompanied with mutations of the amino acid sequences around the cleavage sites. However, the otocephalan clade II enzyme MfHE3 did not cleave the mid-ZPd site of the medaka egg envelope during cross-species digestion experiments (Figure 7D). Furthermore, MfHE3 was inefficient at cleaving the peptide designed from the medaka mid-ZPd site (0.002 μg peptide · mg enzyme^−1^ · 30 min^−1^). Our results suggest that both the substrate and the substrate specificity of the clade II enzyme was altered during the evolution of Euteleostei to gain the mid-ZPd site.

### The driving force of neofunctionalization

The third round of whole genome duplication (3R-WGD) that occurred in the teleost ancestor produced a large number of duplicated genes [32,33]. Subsequent to this, hatching enzyme genes were subject to several additional lineage-specific duplication events [16]. This series of duplication events has resulted in a high level of hatching enzyme gene diversity, and is a driving force of neofunctionalization. Interestingly, we have reported that the hatching enzyme genes of teleosts frequently lost their introns during the evolution [15]. Chromosomal synteny around clade II gene was conserved in euteleosts, such as medaka, three-spined stickleback, and *Tetraodon*. However, the synteny around clade I gene was not conserved in euteleosts. Furthermore, conserved synteny was not observed between euteleostean clade I genes and clade II genes, or between otocephalan clade I genes (zebrafish, ZHE1) and euteleostean clade I genes. The ZHE1 gene cluster is located at inton 17 of the aox2 gene, suggesting the genes were translocated by retrotransposable element (s) [14]. Such evolutionary events accompanied with a large structural change in the hatching enzyme genes likely contributed to the diversification of the hatching enzyme genes.

## Conclusions

We inferred the neofunctionalization process of the clade II hatching enzyme (Figure 1C). After duplication of the gene, the ancestral clade II gene has gained N-ZPd cleavage activity in the lineage of common ancestors of Euteleostei and Otocephala. Subsequently, acquisition of cleavage activity at the mid-ZPd site and loss of cleavage activity in the N-terminal region has occurred during the evolution of Euteleostei, but not Otocephala. Therefore, the addition of the two cleavage sites is presumed to be the result of independent evolutionary events. This hypothesis is supported by the egg envelope structure dependent cleavage of N-ZPd and mid-ZPd sites as follows. The euteleostean clade II enzyme is inefficient at digesting the intact egg envelope, but is able to cleave at two sites in the clade I enzyme-mediated swollen egg envelope. Therefore, MHCE-mediated cleavage renders the egg envelope structure loosened and provides MLCE access to the cleavage sites. Furthermore, the cleavage of the N-ZPd site is a prerequisite for cleavage of the mid-ZPd site. The nine-spined stickleback is unique in that the species possesses two types of clade II genes, LCEα and LCEβ, that share the original function, i.e., subfunctionalization. LCEα cleaves the N-ZPd site but not the mid-ZPd site, whereas LCEβ cleaves only the mid-ZPd site. LCEβ cannot solubilize the HCE-mediated swollen egg envelope, but solubilizes the swollen egg envelope treated by HCE and LCEα, which digest the sites at the N-terminal region and N-ZPd site [34]. These observations suggest that the stepwise cleavage of the egg envelope proteins, N-terminal region, N-ZPd site, and the mid-ZPd site, leads to complete solubilization. Therefore, the neofunctionalization of the euteleostean clade II gene occurred during two independent evolutionary events, the first resulted in the addition of the N-ZPd site and the second the addition of the mid-ZPd site.

Our enzymological experiments suggest that the change in substrate was one of the most important factors for both the addition of the mid-ZPd site and the loss of activity at the N-terminal region. The peptide digestion experiment revealed that the cleavage specificity of clade II and clade I enzymes were the same in some instances, but also differed for some sites. This implies that the specificity of the clade II enzyme was changed in a gradual process during evolution after the duplication. A drastic change in specificity during evolution would likely have resulted in loss of biological function of the gene, followed by loss of the gene itself. Astacin family proteases suggested to have broad substrate specificity [35,36] and fish hatching enzymes are no exception [23,37,38]. Therefore, after the duplication, the clade II enzymes have apparently shifted their substrate preference from that of the ancestral enzyme, while retaining some of the cleavage specificity of clade I enzymes. The creation of a new cleavage sites seems to have been achieved by a mutation in the ancestral sequence at the corresponding sites N-ZPd and mid-ZPd which resulted in a sequence match for the substrate preference of clade II enzymes. Following this change, the role sharing of clade I and II enzymes in egg envelope digestion have been established during the co-evolutional process of the enzymes and their respective cleavage sites. Thus, the two-enzymes system for solubilization of the egg envelope has been conserved in Euteleostei.

Our results suggest that neofunctionalization of genes occurs when a gene product creates a new interaction with another product, and the genes successfully co-evolve. Our results provide an example of the process of appearance of a neofunctional gene resulting from coordination of a mutation that occurred in two related genes.

## Methods

### Ethics statement

The experiments were performed in accordance with Law for the Humane Treatment and Management of Animals in Japan [39]. The law was enacted in 1973, amended in 1999, and coordinated with the Institutional Animal Care and Use Committee (IACUC) protocols [40].

### Egg envelopes

Milkfish, *C. chanos*, ovary samples were provided by the Aquaculture Department of Southeast Asia Fisheries Development Center (SEAFDEC/AQD) in the Philippines. For sacrifice of the milkfish, mature female milkfish were spinalized under anesthetized using 2-phenoxy-ethanol. Ovary with unfertilized egg envelopes was homogenized in 20 mM Tris–HCl (pH 8.0) containing 0.13 M NaCl, 5 mM EDTA, and 5 mM iodoacetic acid. The envelopes were washed several times with the same buffer. The isolated unfertilized egg envelopes were stored at −20°C until analysis. To isolate the milkfish fertilized egg envelope, pre-hatch embryos (9–11 h after fertilization) were crushed in seawater. The crushed egg envelopes were washed several times with seawater to completely remove the yolk proteins and cell debris. The fertilized egg envelopes of medaka *Oryzias latipes* were isolated from eggs 5 d after fertilization, and washed with distilled water using the procedure described above. The isolated unfertilized and fertilized egg envelopes were used for digestion experiments.

### Generation of recombinant hatching enzymes

The fragments containing the mature enzyme portion of each MfHE cDNA were individually transferred into the expression vector pET3c (Promega, Madison, WI). The recombinant proteins were harvested as the inclusion bodies [23]. To facilitate refolding, the inclusion bodies were dissolved in 50 mM Tris–HCl (pH 8.0) containing 8 M urea and 0.1 M 2-mercaptoethanol. After incubation at 37°C for 30 min, the mixture was diluted 1000-fold with 50 mM Tris–HCl (pH 8.0) containing 0.8 M arginine, 1 mM GSH, 0.1 mM GSSG, 5 μM ZnSO_4_, and 0.01% Briji 35, and allowed to stand for 2 days at 4°C. The solution was then dialyzed against 50 mM Tris–HCl (pH 8.0) containing 0.5 M NaCl and 0.01% Briji 35. The recombinant MfHEs were purified by Ni-NTA Superflow chromatography (Qiagen, Valencia, CA, USA) or concentrated by an Amicon Ultra-15 (Millipore Co., Billerica, MA). Recombinant zebrafish hatching enzyme, rZHE1, was prepared according to a method described previously [23].

### Estimation of caseinolytic activity

The caseinolytic activity of the hatching enzymes was measured in a 375 μL reaction mixture consisting of 83 mM Tris–HCl (pH 8.0) and 3.3 mg/mL of casein and the enzyme. The reaction mixture was incubated for 30 min at 30°C. After the reaction was stopped by adding 125 μL of 20% perchloric acid, the mixture was allowed to stand in an ice-cold water bath for 10 min, then centrifuged at 18,500 g for 5 min at 4°C. The absorbance of the supernatant was measured at 280 nm.

### Zymography of rMfHE1 and rMfHE3

The bands for active rMfHE1 and rMfHE3 were observed by casein zymography. Briefly, the recombinant proteins were electrophoresed on a sodium dodecyl sulfate-polyacrylamide gel containing 0.1% casein. The refolded rMfHE1 or rMfHE3 samples were mixed with an equal volume of 2 × SDS-sample buffer without 2- mercaptoethanol, at 80°C for 30 min. After electrophoresis, the gel was washed in 20 mM Tris–HCl (pH 8.0) containing a 2% Triton-X 100 for 1 h at 30°C, then incubated in 20 mM Tris–HCl (pH 8.0) and 0.1 mM ZnSO_4_ at 30°C overnight with gentle agitation. The gel was stained with Coomassie Brilliant Blue R-250 (Sigma, St. Louis, MO) and then destained.

### Egg envelope digestion experiments

Eight to ten unfertilized and fertilized egg envelopes were incubated in 20 μL of 25 mM Tris–HCl (pH 8.0), 0.5 M NaCl, and an appropriate amount of enzyme at 30°C. Because the refolding efficiencies of mutant recombinant proteins differed from sample to sample, the amount of active enzyme added to the reaction mixture was adjusted based on the caseinolytic activity. The same amount of caseinolytic activity was added to the reaction mixture (0.075 Δ_280_ · 30 min^−1^ for digestion of unfertilized egg envelopes and 0.3 Δ_280_ · 30 min^−1^ for digestion of fertilized egg envelopes). After incubation, the digested egg envelopes were photographed by Leica MZFLIII stereo microscope (Leica, Solms, Germany), and subjected to SDS-PAGE.

### Determination of N-terminal sequences of the digests

Egg envelopes were analyzed by SDS-PAGE, and electrically blotted onto a PVDF membrane (Hybond-P, GE Healthcare UK Ltd., Buckinghamshire, England). After staining with CBB, the band was cut out and submitted for protein sequencing (Procise 491HT, Applied Biosystems, Foster City, CA, USA).

### Synthetic peptide cleavage activity

The synthetic peptides spanning 10–11 amino acids were designed based on the sequence around the cleavage sites of several fish hatching enzymes. For ZHE1, MHCE, and MLCE, a 25 μL reaction mixture consisting of 50 mM Tris–HCl (pH 8.0), 2.5 nmol peptide, and an appropriate amount of enzyme was incubated at 30°C for 30 min. For MfHE1 and MfHE3, a 25 μL reaction mixture consisting of 50 mM Tris–HCl (pH 8.0), 0.5 M NaCl, 2.5 nmol peptide, and an appropriate amount of enzyme was incubated at 30°C for 30 min. After the reaction was stopped by the addition of 2.5 μL of 0.1 M EDTA, the reaction mixtures were applied to a C18 column (YMC Co. Ltd., Kyoto, Japan) on an HPLC system equilibrated with 0.1% TFA and eluted with a linear gradient of 0–60% acetonitrile in 0.1% TFA. The activity was calculated from the ratio of the peak areas of the digested and undigested peptides. The values were expressed as the specific activity of the purified enzymes (μg peptide · mg enzyme^−1^ · 30 min^−1^).

### Purification of medaka hatching enzyme

Hatching enzymes from medaka (MHCE and MLCE) were purified from hatching liquids (culture medium of embryos immediately after hatching) according to a method described previously [37,38].

## Abbreviations

MfHE: Milkfish hatching enzyme
rMfHE: Recombinant milkfish hatching enzyme
ZP: Zona pellucida
N-ZPd: N-terminus of ZP domain
mid-ZPd: Middle of ZP domain
HCE: High choriolytic enzyme
LCE: Low choriolytic enzyme
MHCE: Medaka high choriolytic enzyme
MLCE: Medaka low choriolytic enzyme
ZHE1: Zebrafish hatching enzyme 1
FHCE: *Fundulus* low choriolytic enzyme
ChgH: Choriogenin H
ChgHm: Choriogenin H minor

## References

[1] Inoue K, Naruse K, Yamagami S, Mitani H, Suzuki N, Takei Y (2003). Four functionally distinct C-type natriuretic peptides found in fish reveal evolutionary history of the natriuretic peptide system. Proc Natl Acad Sci U S A.

[2] Thompson A, Vo D, Comfort C, Zakon HH (2014). Expression evolution facilitated the convergent neofunctionalization of a sodium channel gene. Mol Biol Evol.

[3] Han L, Monné M, Okumura H, Schwend T, Cherry AL, Flot D, Matsuda T, Jovine L (2010). Insights into egg coat assembly and egg-sperm interaction from the X-ray structure of full-length ZP3. Cell.

[4] Jovine L, Qi H, Williams Z, Litscher E, Wassarman PM (2002). The ZP domain is a conserved module for polymerization of extracellular proteins. Nat Cell Biol.

[5] Darie CC, Janssen WG, Litscher ES, Wassarman PM (2008). Purified trout egg vitelline envelope proteins VEβ and VEγ polymerize into homomeric fibrils from dimers *in vitro*. Biochim Biophys Acta.

[6] Litscher ES, Janssen WG, Darie CC, Wassarman PM (2008). Purified mouse egg zona pellucida glycoproteins polymerize into homomeric fibrils under non-denaturing conditions. J Cell Physiol.

[7] Yasumasu S, Kawaguchi M, Ouchi S, Sano K, Murata K, Sugiyama H, Akema T, Iuchi I (2010). Mechanism of egg envelope digestion by hatching enzymes, HCE and LCE in medaka, *Oryzias latipes*. J Biochem.

[8] Yasumasu S, Yamada K, Akasaka K, Mitsunaga K, Iuchi I, Shimada H, Yamagami K (1992). Isolation of cDNAs for LCE and HCE, two constituent proteases of the hatching enzyme of *Oryzias latipes*, and concurrent expression of their mRNAs during development. Dev Biol.

[9] Bond JS, Beynon RJ (1995). The astacin family of metalloendopeptidases. Protein Sci.

[10] Yamagami K (1972). Isolation of a choriolytic enzyme (hatching enzyme) of the teleost, *Oryzias latipes*. Dev Biol.

[11] Yasumasu S, Iuchi I, Yamagami K (1988). Medaka hatching enzyme consists of two kinds of proteases which act cooperatively. Zool Sci.

[12] Yasumasu S, Mao KM, Sultana F, Sakaguchi H, Yoshizaki N (2005). Cloning of a quail homologue of hatching enzyme: its conserved function and additional function in egg envelope digestion. Dev Genes Evol.

[13] Katagiri C, Maeda R, Yamashika C, Mita K, Sargent TD, Yasumasu S (1997). Molecular cloning of Xenopus hatching enzyme and its specific expression in hatching gland cells. Int J Dev Biol.

[14] Kawaguchi M, Yasumasu S, Hiroi J, Naruse K, Suzuki T, Iuchi I (2007). Analysis of the exon–intron structures of fish, amphibian, bird and mammalian hatching enzyme genes, with special reference to the intron loss evolution of hatching enzyme genes in Teleostei. Gene.

[15] Kawaguchi M, Hiroi J, Miya M, Nishida M, Iuchi I, Yasumasu S (2010). Intron-loss evolution of hatching enzyme genes in Teleostei. BMC Evol Biol.

[16] Kawaguchi M, Yasumasu S, Hiroi J, Naruse K, Inoue M, Iuchi I (2006). Evolution of teleostean hatching enzyme genes and their paralogous genes. Dev Genes Evol.

[17] Nelson JS (2006). Fishes of the world.

[18] Hiroi J, Maruyama K, Kawazu K, Kaneko T, Ohtani-Kaneko R, Yasumasu S (2004). Structure and developmental expression of hatching enzyme genes of the Japanese eel *Anguilla japonica*: an aspect of the evolution of fish hatching enzyme gene. Dev Genes Evol.

[19] Sano K, Kawaguchi M, Yoshikawa M, Kaneko T, Tanaka T, Iuchi I, Yasumasu S (2011). Hatching enzyme of Japanese eel *Anguilla japonica* and the possible evolution of egg envelope digestion mechanism. FEBS J.

[20] Lee KS, Yasumasu S, Nomura K, Iuchi I (1994). HCE, a constituent of the hatching enzymes of *Oryzias latipes* embryos, releases unique proline-rich polypeptides from its natural substrate, the hardened chorion. FEBS Lett.

[21] Jovine L, Darie CC, Litscher ES, Wassarman PM (2005). Zona pellucida domain proteins. Annu Rev Biochem.

[22] Kawaguchi M, Yasumasu S, Shimizu A, Sano K, Iuchi I, Nishida M (2010). Conservation of the egg envelope digestion mechanism of hatching enzyme in euteleostean fishes. FEBS J.

[23] Sano K, Inohaya K, Kawaguchi M, Yoshizaki N, Iuchi I, Yasumasu S (2008). Purification and characterization of zebrafish hatching enzyme: An evolutionary aspect of the mechanism of egg envelope digestion. FEBS J.

[24] Iuchi I, Ha CR, Sugiyama H, Nomura K (1996). Analysis of chorion hardening of eggs of rainbow trout, *Onchorhynchus mykiss*. Dev Growth Differ.

[25] Sugiyama H, Iuchi I (2000). Molecular structure and hardening of egg envelope in fish. Recent Res Dev Comp Biochem Physiol.

[26] Sano K, Kawaguchi M, Watanabe S, Nagakura Y, Hiraki T, Yasumasu S (2013). Inferring the evolution of teleostean zp genes based on their sites of expression. J Exp Zool.

[27] Sano K, Kawaguchi M, Yoshikawa M, Iuchi I, Yasumasu S (2010). Evolution of the teleostean zona pellucida gene inferred from the egg envelope protein genes of the Japanese eel, *Anguilla japonica*. FEBS J.

[28] Wallace RA, Jared DW (1969). Studies on amphibian yolk. 8. The estrogen-induced hepatic synthesis of a serum lipophosphoprotein and its selective uptake by the ovary and trasformation into yolk platelet proteins in *Xenopus laevis*. Dev Biol.

[29] LaFleur GJ, Raldúa D, Fabra M, Carnevali O, Denslow N, Wallace RA, Cerdà J (2005). Derivation of major yolk proteins from parental vitellogenins and alternative processing during oocyte maturation in *Fundulus heteroclitus*. Biol Reprod.

[30] Stehr CM, Hawkes JW (1979). The comparative ultrastucture of egg membrane and associated pore structure in the starry flounder, *Platichthys stellatus* (Pallas), and pink salmon, *Oncorynchus gorbuscha* (Walbaum). Cell Tissue Res.

[31] Sugiyama H, Yasumasu S, Murata K, Iuchi I, Yamagami K (1998). The third egg envelope subunit in fish: cDNA cloning and analysis, and gene expression. Dev Growth Differ.

[32] Chiu CH, Dewar K, Wagner GP, Takahashi K, Ruddle F, Ledje C, Bartsch P, Scemama JL, Stellwag E, Fried C, Prohaska SJ, Stadler PF, Amemiya CT (2004). Bichir HoxA cluster sequence reveals surprising trends in ray-finned fish genomic evolution. Genome Res.

[33] Naruse K, Tanaka M, Mita K, Shima A, Postlethwait J, Mitani H (2004). A medaka gene map: the trace of ancestral vertebrate proto-chromosomes revealed by comparative gene mapping. Genome Res.

[34] Kawaguchi M, Takahashi H, Takehana Y, Naruse K, Nishida M, Yasumasu S (2013). Sub-functionalization of duplicated genes in the evolution of nine-spined stickleback hatching enzyme. J Exp Zool.

[35] Wolz RL, Bond JS (1990). Phe 5 (4-nitro)- bradykinin: a chromogenic substrate for assay and kinetics of the metalloendopeptidase meprin. Anal Biochem.

[36] Wolz RL, Harris RB, Bond JS (1991). Mapping the active site of meprin-A with peptide substrates and inhibitors. Biochemistry.

[37] Yasumasu S, Iuchi I, Yamagami K (1989). Purification and partial characterization of high choriolytic enzyme (HCE), a component of the hatching enzyme of the teleost, *Oryzias latipes*. J Biochem.

[38] Yasumasu S, Iuchi I, Yamagami K (1989). Isolation and some properties of low choriolytic enzyme (LCE), a component of the hatching enzyme of the teleost, *Oryzias latipes*. J Biochem.

[39] **Investigative Committee on the Law for the Humane Treatment and Management of Animals.** In *Explanatory Handbook on the Law (text in Japanese).* Tokyo: Seirin Shoin; 2001 [http://www.alive-net.net/english/en-law/L2-full-text.html]

[40] **Institutional animal care and Use committee.** [http://iacuc.usc.edu/]

